# Supplementary material for: Prevalence and predictors of transfusion-transmitted infections among blood donor types at a teaching hospital in Ghana: Implications for haemovigilance
Source: PLoS One. 2025 Oct 31;20(10):e0335544. doi: 10.1371/journal.pone.0335544 (PMC12578228; doi:10.1371/journal.pone.0335544)
Supplement: S1 Table — (DOCX) [file pone.0335544.s001.docx]

**S1 Table. Specifications and performance metrics of screening kits used for the detection of transfusion-transmitted infections (TTIs).**

| **Test Type** | **Kit Name** | **Manufacturer** | **Sensitivity (%)** | **Specificity (%)** |
| --- | --- | --- | --- | --- |
| HBV (RDT) | Standard Q® HBsAg | SD Biosensor, Inc. (Republic of Korea) | 100.0* | 100.0* |
| HCV Ab (RDT) | Standard Q® HCV Ab | SD Biosensor, Inc. (Republic of Korea) | 100.0 | 97.2 |
| HIV (RDT) | First Response® HIV 1-2.0 card test (Antibodies) | Premier Medical Corporation (India) | 100.0# | 100.0# |
| Syphilis (RDT) | Advanced Quality™ One-Step Anti-TP (TP/syphilis) | InTec PRODUCTS, China | 97.5 | 98.5 |
| HBV (ELISA) | Fortress Diagnostics HBsAg | Fortress Diagnostics Ltd (United Kingdom) | Not stated | 99.8 |
| HCV (ELISA) | Anti-HCV (4th Generation) | Fortress Diagnostics Ltd (United Kingdom) | Not stated | 99.6 |
| HIV (ELISA) | Fortress Diagnostics 4th Gen HIV 1 & 2 (Ag/Ab) | Fortress Diagnostics Ltd (United Kingdom) | Not stated | Not stated |
| Syphilis (ELISA) | Syphilis Treponema pallidum Antibody | Fortress Diagnostics Ltd (United Kingdom) | Not stated | 99.9 |

Performance characteristics (sensitivity and specificity) as provided by kit manufacturers. ELISA tests were performed on a ChemWell® FUSION automated analyser (Awareness Technologies, USA). RDT = Rapid Diagnostic Test; ELISA = Enzyme-Linked Immunosorbent Assay; HBV = Hepatitis B Virus; HCV = Hepatitis C Virus; HIV = Human Immunodeficiency Virus.
*Manufacturer-claimed performance using CLIA analyser as reference.
#Manufacturer-claimed performance using ELISA as reference.
