# Supplementary material for: Prevalence and predictors of transfusion-transmitted infections among blood donor types at a teaching hospital in Ghana: Implications for haemovigilance
Source: PLoS One. 2025 Oct 31;20(10):e0335544. doi: 10.1371/journal.pone.0335544 (PMC12578228; doi:10.1371/journal.pone.0335544)
Supplement: S2 Table — (DOCX) [file pone.0335544.s002.docx]

**S2 Table. Relationship between** **sociodemographic characteristics and donation site among** **potential blood donors (N = 10,152).**

|  | **Off-site donors N (%)** | **On-site donors N (%)** | **Total, N (%)** | **P value** |
| --- | --- | --- | --- | --- |
| **Total, N** | ***N=3523*** | ***N=6629*** | ***N =*** ***10152*** | **<0.001** |
| **Age group** |  |  |  | **<0.001** |
| 16 - 20 years | 2282 (64.8%) | 501 (7.6%) | 2783 (27.4%) |  |
| 21 - 30 years | 947 (26.9%) | 3291 (49.6%) | 4238 (41.7%) |  |
| 31 - 40 years | 192 (5.4%) | 2097(31.6%) | 2289 (22.5%) |  |
| 41–60 years | 102 (2.9%) | 740 (11.2%) | 842 (8.3%) |  |
| **Gender** |  |  |  | **<0.001** |
| Females | 1446 (41.0%) | 565 (8.5%) | 2011 (19.8%) |  |
| Males | 2077 (59.0%) | 6064 (91.5%) | 8141 (80.2%) |  |
| **D. History** |  |  |  | **<0.001** |
| First-time D. | 2641 (75.0%) | 2830(42.7%) | 5471 (53.9%) |  |
| Repeat donors | 882 (25.0%) | 3799 (57.3%) | 4681 (46.1%) |  |
| **Occupation** |  |  |  | **<0.001** |
| Student | 2983 (84.7%) | 801 (12.1%) | 3784 (37.3%) |  |
| Worker | 517 (14.7%) | 5310 (80.1%) | 5827 (57.4%) |  |
| Unemployed | 9 (0.3%) | 304 (4.6%) | 313(3.1%) |  |
| Others * | 14 (0.4%) | 208 (3.2%) | 214 (3.2%) |  |
| **Overall TTI^#^** |  |  |  | **<0.001** |
| Positive | 378 (10.7%) | 1297 (19.6%) | 1675 (16.5%) |  |
| Negative | 3145 (89.3%) | 5332 (80.4%) | 8473 (83.5%) |  |

The sociodemographic characteristics of potential blood donors, stratified by donation site (Off-site (voluntary mobile donors only) vs. On-site (voluntary walk-in and replacement donors). % =percentage

*Others include apprentices, retirees, prisoners, and individuals who did not specify their occupation. D. = Donors or Donation, TTI = Transfusion-transmitted infections.

# Overall TTIs positivity is based on donors reactive/positive for at least one infectious marker.
