# Supplementary material for: Prevalence and predictors of transfusion-transmitted infections among blood donor types at a teaching hospital in Ghana: Implications for haemovigilance
Source: PLoS One. 2025 Oct 31;20(10):e0335544. doi: 10.1371/journal.pone.0335544 (PMC12578228; doi:10.1371/journal.pone.0335544)
Supplement: Attachment — Submitted filename: Response to Reviewers.pdf [file pone.0335544.s005.pdf]

Editor-in-Chief

PLOS ONE

30 June 2025

Dear Editor,

Re: Revised manuscript submission manuscript number: PONE-D-25-18662

Title: Prevalence and predictors of transfusion-transmitted infections among blood donor types at a teaching hospital in Ghana: implications for haemovigilance.

We are sincerely grateful to the reviewers for their time, insightful comments, and constructive feedback, which have contributed significantly to improving the quality and rigour of our manuscript. We also thank the Editor for the opportunity to revise our work for possible publication in PLOS ONE.

The manuscript has been extensively revised to address all comments, and our responses follow each point.

We hope the revised version meets the requirements for publication in *PLOS ONE*, and we are grateful for the opportunity to resubmit.

Thank you for your consideration.

Yours faithfully,

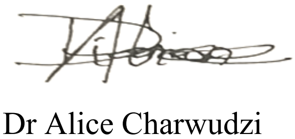

Dr Alice Charwudzi

Corresponding author

Reviewer comments and author responses

REVIEWER #1

Comment: While the article is well-written and presents engaging statistical analyses that captured my interest, it contains some shortcomings and errors, particularly in the discussion section, which I will address separately.

Response: We thank the reviewer for the constructive comment.

Abstract:

Comment: The methods section in the abstract and the article's main text could benefit from additional information or examples of RDT tests to clarify the concept for readers.

Response: We are grateful to the esteemed reviewer for this comment. This additional information about the RDTs used (e.g., Determine™ for HIV, SD Bioline™ for HBsAg and HCV, and yphilis Rapid Test Strip™) has been provided.

Comment: In the author summary section, it would be helpful for the author to explain concisely the rationale behind emphasising NAT over RDTs.

Response: We expanded the author summary to briefly explain that NAT offers improved sensitivity, particularly during the early window period of infection, but remains cost-prohibitive in low-resource settings. Thus, ELISA is presented as a more feasible alternative for improving blood safety.

Introduction:

Comment: The text does not provide statistics on the prevalence of the mentioned viruses or TTIs within the geographical area of the study, which would enhance readers' understanding. Additionally, including a global prevalence statistic would offer a more comprehensive comparative perspective, especially since the text claims that the prevalence in the studied area is higher than the worldwide average.

Response: Thank you for these comments. The entire introduction section has been extensively modified to reflect all the concerns raised, in addition to provided more information to make it more comprehensible.

Comment: If prior studies have been conducted on the prevalence of TTIs in this geographical area or similar regions with comparable socio-economic conditions, briefly referencing those statistics would be beneficial. This allows for examining the trends in TTI prevalence and the global trend over time.

Response: We are grateful for these comments. More information with regards to this topic in our geographical area or similar regions has been included and referenced accordingly, including Tetteh et al. (2018) and Okoroiwu et al. (2021). This enhances the reader's understanding of the local context.

Materials and Methods:

Comment: The section on ethical approval typically appears at the end of the document, preceding the references section..

Response: Thank you for the observation. As per journal format, the ethical issues or considerations have been moved to the end of the methodology section in agreement with journal guidelines.

Comment: In line 126, the phrase "The study included data from 10,152 blood" should be revised to replace "blood" with "donated blood" or "blood sample."

Response: The entire methodology section has been extensively modified to reflect all the concerns raised. The sentence now reads: "The study included data from 10,152 potential blood donors…

Comment: Could you clarify the meaning of "motivational incentives" or provide some examples?

Response: We now clarify that "motivational incentives" refer to post-donation refreshments and small souvenir items (e.g., exercise books) provided to voluntary donors to encourage participation.

Comment: The parentheses at the end of line 156 were incorrectly placed and have been removed

Response: The parentheses at the end of line 156 have been removed appropriately.

Results:

Comment: In the Donor Demographics section, information for both donor groups should be consistent and uniform. This means that the percentage figures for either the student group or the age group should be included in lines 167 and 168. Alternatively, "3210" and "5188" should be removed from lines 169 and 170, respectively

Response: Consistency has been ensured in the presentation of the results. All the necessary modifications have been made.

Comment: In tables, the letter N, denoting the number, should be presented in uppercase.

Response: All instances of "n" have been revised to uppercase "N" in all the tables.

Comment: Explanations provided below the tables corresponding to the same table should be formatted in a smaller font to enhance clarity and distinguish them from the main text.

Response: We are grateful for the comment. We reformatted all table footnotes according to the journal's guidelines.

Comment: In Table 4, the font for the numbers must be consistent throughout.

Response: The font in Table 3 (previously Table 4) has been reviewed and standardised to ensure consistency across all numeric entries, and the font size has been maintained at 11 for consistency.

Comment: In the Co-Infections section, no statistical analysis was performed to compare the two donor groups regarding the presence of a significant difference. What is the reason for this?

Response: The necessary associations to compare the two donor groups have been made and added to the write-up at the co-infections section. The statistical comparison of co-infection rates between donor groups yielded a non-significant result (p=0.648).

Discussion:

Comment: Although the focus of the text, according to the explanations at the end of the Introduction section, is to examine the status of TTIs and their prevalence in different types of blood donors and to emphasize the weakness of RDTs for identifying infected donors (these issues are well addressed in the Discussion section), the main problem with this article is the Discussion section.

Response: We appreciate the reviewer for these comments. From the comments it is a bit confusing what the esteemed review wanted to convey but we assumed that it was relating to the improvement of the discussion relating to TTIs and their prevalence in different types of blood donors, which have been duly improved.

Comment: In the Results section, this study thoroughly examines HBV, HCV, HIV, and syphilis, with well-organized data presented in the tables. This approach leads the reader to anticipate speculation and discussion of these topics in the subsequent Discussion section. Even in some cases, the lack of significance of the difference between the two groups under study is worthy of discussion and speculation (for example, Table 2 of the HIV section in the Donation History section), but in the Discussion section, there is no mention of these data and no discussion is raised in this regard. It would be better to have a detailed discussion in this section regarding the data obtained from these infectious agents.
Response: We agree with the reviewer and have expanded the discussion to interpret the findings for each TTI, syphilis, HBV, HCV, and HIV, including cases where no statistically significant difference was observed, under the heading “The prevalence of syphilis, HBV, HCV and HIV” to improve the discussion section.

Images and diagrams:

Comment: The quality of images 2 and 4 is currently low; it is advisable to provide improved versions. Additionally, the explanatory text overlaps with the image borders in certain sections of the flowchart or images, making it challenging to read. This issue should be addressed. Furthermore, for the text related to images 2 and 3, use the term "Figure" instead of "Fig" to ensure consistency across all images.
Response: We have improved the image quality for Figures 2 and 4 with high-resolution versions, and the image quality meets the journal requirement when checked on NAAS (https://ngplosjournals.pagemajik.ai/artanalysis). We have also improved spacing in the flowchart to eliminate text overlap, and standardised terminology throughout the manuscript to use “Fig” as per journal style.

Comment: The flowchart is also recommended to indicate the total number of subjects studied (10,152). At each stage, it would be beneficial to specify the number of subjects who tested positive for TTI and are no longer eligible for donation. This addition will enhance the reader's

understanding of a significant aspect of the article's purpose and provide a more precise context before delving into the full text.

Response: The revised flowchart now begins with the full donor cohort (N = 10309) and details the number of TTI-positive donors excluded at each stage. This enhances transparency and guides the reader through the donor inclusion process.

REVIEWER #2

Comment 1- In this manuscript: you wrote (Our)!!! The rule of manuscript writing is to avoid using (Our). So you should delete (Our), use the formal scientific words (This study or The current study or The present study).

Response:

Thank you for your observation. We have revised the manuscript thoroughly and replaced all instances of the term "our" with more appropriate and formal alternatives such as "this study" or "the current study," as recommended.

Comment 2: You wrote (We) several times!!! The rule of manuscript writing is to avoid using (We). So you should delete (We), use the formal scientific words (This study or The current study or The present study).

Response: We appreciate this suggestion. We have carefully revised the manuscript and replaced all occurrences of "we" with formal, third-person expressions, such as "the study" or "the current study," per standard scientific writing conventions.

REVIEWER #3

Comment: This is a well-structured and relevant study that explores the prevalence and predictors of transfusion-transmitted infections (TTIs) among different blood donor types in a teaching hospital in Ghana. The topic is extremely relevant, particularly when considering blood safety in environments with limited resources. The findings promote the adoption of more sensitive screening methods and the promotion of voluntary blood donation, both of which are important for public health. While the manuscript is generally strong in scope and presentation, a few areas need clarification and revision to meet PLOS ONE's standards,

especially regarding methodological transparency, statistical presentation, and some grammatical and formatting improvements.

Response: We appreciate the reviewer for the positive feedback on our manuscript and the constructive suggestions to help improve the manuscript.

Comment: The study addresses a significant public health issue in a resource-limited setting where transfusion safety is critical. 2. The sample size is large (15,683 donors), which enhances the statistical precision and reliability of the results. 3. A comprehensive picture of transfusion risk is provided by the analysis, which covers several TTI types (HIV, HBV, HCV, and syphilis). 4. By using multivariable logistic regression, the evaluation of predictors becomes strengthened. 5. The authors adequately explain the consent procedure and offer ethical approval.

Responses: We appreciate the reviewer for the positive feedback on our manuscript, which is very encouraging.

Major issues:

Abstract

Comment 1:

Comment 1: Donor categories lack clarity: "Voluntary walk-ins" and "voluntary mobiles" are two examples of terms that are not well defined. It could be defined more clearly for an international audience.

Response: The methodology section has been extensively revised to provide more clarity on the donor categories. Specifically, “voluntary walk-ins” refer to individuals who donate at the fixed-site blood bank without external mobilisation, while “voluntary mobile donors” are individuals recruited through outreach drives at schools, churches, or public locations.

Comment 2: An incomplete explanation of the testing procedures. The number of donors examined using RDTs alone versus RDTs with ELISA, as well as the conditions under which ELISA was employed, are unknown. This makes the sensitivity gap (7.4%) reported in the results less interpretable.

Response: We are grateful for this comment. The methodology section has been extensively revised to provide more context on the proportion of donors tested with RDT only versus RDT and ELISA for clarity. More details have also been added to the flow chart and supplementary Table 1 (S1 Table) to aid with the clarification.

Comment 3: Some statistical terms (e.g., adjusted odds ratios) are used without full context (confidence intervals are missing).
Response: 95% confidence intervals have now been added to the adjusted odds ratios.

Comment 4: I recommend that the authors define all abbreviations at first use in the abstract—for example, "HBV (hepatitis B virus)," "HCV (hepatitis C virus)," and "RDTs (rapid diagnostic tests)"—to improve clarity for readers unfamiliar with these terms,
Response: Done. All abbreviations used in the abstract and throughout the manuscript are now defined at first mention.

Comment 5: The mention that "ELISA confirmed an additional 7.4% of TTI cases among RDT non-reactive donors" is unclear as to additional detection by ELISA. Does this rate apply to RDT-negative donors, all tested donors, or 7.4% of all cases?
Response: We are grateful for the comment. This is an additional detection percentage, which is actually 7.3% (calculation error), obtained after retesting of non-reactive (negative) RDT samples using ELISA. The explanation has been added to the write-up under "Performance for screening methods" of the results section and the flow chart.

Comment 6: Although it is strongly advised, nucleic acid testing (NAT) is not contextualized. The financial, logistical, and technical viability of NAT in low-resource environments—all crucial for such a recommendation—is not discussed.
Response: We have added a sentence to the Author Summary and Discussion noting that while NAT is ideal, its use is constrained in low-resource settings like Ghana due to financial and infrastructural limitations. ELISA is thus presented as a more feasible alternative.

Comment 7: To improve the manuscript's accessibility and searching, I propose expanding the keywords by using more precise and standardized terminology such "ELISA," "repeat donors," and "risk factors."

Response: Keywords have been modified to improve discoverability and comply with Medical Subject Headings (MESH).

Introduction

Comment 1: The increasing incidence of transfusion-transmitted infections (TTIs) is discussed in the introduction, but it is unclear what exact requirement this study aims to fulfill, especially regarding the uniqueness or impact of comparing donor types in the Ghanaian circumstances.
Response: We revised the Introduction to state that the study aims to fill a gap in understanding the differential risk of TTIs among donor types (voluntary vs. replacement) in the context of Ghana, where replacement donation is still widely practised. The rationale for comparing these groups is now made explicit.

Comment 2: Background information on Ghana's blood donation system, such as laws governing replacement vs. voluntary donations, current hemovigilance procedures, or the usage of screening technology, is lacking and is crucial for establishing the study's relevance.
Response: We added a paragraph summarising Ghana’s current blood donation policies, noting that:

- Ghana’s blood donation system is largely governed by the National Blood Service Act,
- Both voluntary and replacement donors are used due to supply constraints.
- Haemovigilance efforts are evolving, but a national donor deferral registry and unified data system are lacking.
- RDTs remain the mainstay of screening in most centres, with limited use of ELISA or NAT due to resource constraints.

Comment 3: Some sentences state well-known facts, such as that TTIs pose a threat to public health, without providing recent, pertinent citations or making a clear connection to the study issue.
Response: The entire manuscript has been thoroughly perused, and significant statements made have been duly cited and referenced.

Comment 4: Uncertain Rationale for Comparing RDT with ELISA: The limitations of RDTs in practical blood screening, which would support the focus, are not sufficiently addressed in the justification for evaluating RDT performance in comparison to ELISA.

Response: We are grateful for this comment. We wanted to find the performance effectiveness of RDT usage in detecting TTIs since they are the most widely used in Ghana, with their attendant poor sensitivity, making them possibly unreliable. ELISA is a better screening method. These have been stated throughout the manuscript.

Comment 5: The Introduction does not conclude with a direct, clear summary of the study's goals or hypotheses to direct the reader to the Methods and Results.
Response: The concluding part of the introduction has been revised with clear study objectives in bullet format to guide the reader clearly into the methods section.

Methods
Comment 1: You wrote that ethical approval was changed in January 2025, which is in the future. However, your data gathering ended before that, so this is confusing. Check the dates to make sure they match the study's time frame.
Response: There are two approval dates, one was for the main ethical approval, and the second one was an amendment to enable us to finalise all the missing or uncaptured data. The two ethical clearance letters were uploaded as supporting files to the journal.

Comment 2: You didn't explain how you picked which donor records to include. For example, did you use all donors from 2022–2023? What about incomplete records? Clearly say who was included and why.
Response: The eligibility criteria have been updated to improve clarity. An initial error noted in the stipulated period for the available data has been corrected to "records from 1st January 2022 to 31st March 2024 …"

Comment 3: It's not clear if repeat donors were counted more than once. This can make the infection rates look higher or lower than they are. Please, say whether the same person was counted once or every time they donated.
Response: We now explain that repeat donations were treated as separate entries due to the lack of a unified donor identification system. Potential donors are given a different unique identification number every time they present for donation.

Comment 4: You state that ELISA was performed after non-reactive RDTs, which is not the standard procedure. The ELISA tests are most frequently used for positive outcomes; however, it is unclear whether you used the ELISA test for all donors or just a few.
Response: We clarified that the protocol at the testing centre does not confirm RDT reactive (positive) cases by ELISA; they are rather deferred, but RDT non-reactive cases (negative) are confirmed to minimise TTIs among fixed-site donors. Voluntary mobile donors received ELISA only, due to donor mobilisation protocols.

Comment 5: You say you removed duplicates and flagged missing data, but you didn't say how or how much was affected. Please, mention how many records were cleaned or what kinds of data problems you found.
Response: Duplicated data were taken as a single occurrence, so it has already been catered for in the total sample. The missing data portion of the manuscript has been modified to provide more clarity and incorporated into the statistical analysis section.

Comment 6: Terms like "walk-in," "mobile donor," and "replacement donor" are used but not clearly defined. Briefly explain what each type means at the beginning (a small table could help).
Response: We thank the esteemed reviewer for this comment. The suggested definitions have been incorporated throughout the revised manuscript, especially in the methodology section.

Comment 7: You did not explain how you selected variables for logistic regression. Also, there is no mention of how you resolved missing data or whether you tested the reliability of the results. Include this information to help readers realize the reliability of your analysis.
Response: Selected variables for logistic regression were based on the literature and significance in bivariate analysis. The missing data portion of the manuscript has been modified to provide more clarity and incorporated into the statistical analysis section.

Comment 8 Some donors received motivations (such as gifts), whereas others did not. This could affect who comes to give and influence the outcome. Mention this as a potential research limitation or bias. 9. There are minor grammatical errors and imprecise language. Proofread the section thoroughly to ensure it is clear and correct.

Response: The motivational packages are given to voluntary donors. These gifts are not significant enough to attract individual to donate a whole pint of their blood. It is just a token that some individuals may even refuse. Replacement donors donate on demand without any incentives from the blood bank. All grammatical errors and imprecise language have been corrected.

Comment 9: Please include brand information, sensitivity and specificity, and internal quality control techniques for ELISA testing. Additionally, a List of manufacturers' indicators of performance for RDT kits. This is crucial, given the significant difference between RDT and ELISA results.
Response: Brand names, sensitivity/specificity values, and internal quality control methods are now included in the Laboratory testing subsection, with a detailed summary in the current Supplementary Table S1 (S1 Table).

Results
Comment 1: The text contains many of the exact numbers as the tables. Please, only highlight the most essential findings in the text. Allow the tables to reveal the details.
Response: Thank you for this suggestion. The result section has been summarised to highlight the most essential findings.

Comment 2. The results are disorganized, jumping from one topic to another. Use subheadings such as "Donor Characteristics," "TTI Prevalence," "Co-infections,"...etc. to guide the reader.
Response: Thanks for the comments. We have tried to present the results in a more organised fashion as per your constructive suggestions.

Comment 3: The number of co-infections (those who have more than one infection) is uncertain. It's unclear whether any contributors were included twice. Explain exactly how these numbers were counted and whether they overlap.
Response: We now explain that co-infections were counted only once per donor. Individuals with more than one TTI were classified under co-infections, and each case was unique.

Comment 4: In certain areas, the total number of contributors is 10,152, whereas in others it is 8,356. There is no explanation. Explain why some data is missing (for example, not all donors provided blood group information).

Response: Thanks for the comments. In this revised version of the manuscript, the 10,152 represents the total cohort, and the 8,356 represents TTI negative blood group donors whose donated blood was safe for cross-matching, hence their blood groups were retrievable. The manuscript has been revised accordingly.

Comment 5: Some figures are mentioned in the text (such as Figures 2, 3, and 4), but they are not shown or explained in detail. Ensure that all figures are included and clearly defined in the text.

Response: All the figures mentioned in the text were uploaded to the journal as per the journal's guidelines, and their legends were included in the main manuscript. All the figures are now well referenced in the main text. Thank you for the comments.

Comment 6: P-values (e.g., $p < 0.001$) are provided; however, crucial statistics such as odds ratios and confidence intervals are either missing or not explained. Please, include these numbers and explain them in simple terms.

Response: In the revised script, p-values have been reported with their corresponding odds ratios and 95% confidence intervals. The interpretation of the reported odds ratio has been included in the statistical analysis and parts of the results sections.

Comment 7: The tables lack clarity in style, and several headers are unclear. Apply the same structure to all tables and clarify all terminology and abbreviations.

Response: These suggestions have all been addressed.

Comment 8: Although the logistic regression model (Table 4) is presented, the authors do not provide an explanation for their actions. Give a brief explanation of the model's elements and testing methods.

Response: These suggestions have all been addressed in the statistical analysis and result sections. In the revised manuscript, Table 4 is now Table 3.

Comment 9: Depending on the context, the word "TTI" can refer to any infection or a group

of infections. I suggested that the term "overall TTI" be defined precisely and consistently.
Response: The overall TTI has been defined precisely, and other instances where it was used ambiguously have been revised to maintain its original meaning.

Comment 10: The frequency of each infection is reported by the authors; however, they exclude confidence intervals, which indicate how accurate the figures are. I suggested that 95% confidence intervals be included with each percentage.
Response: 95% confidence intervals are now included for all prevalence estimates and odds ratios.

Comment 11. Group differences (students versus employees, for example) are explained without addressing further potential reasons.
Recommendation: When describing these patterns, exercise greater caution and address relevant confounding variables.
Response: The demographic information helped us ascertain the significant predictors of TTIs. The findings showed that most voluntary donors were students with lower TTIs prevalence, making them the safest group to be relied on for voluntary donation.

Comment 12: The Results section should just focus on the findings. However, in some cases, the authors interpret the meaning. Remove any opinions or interpretations to the Discussion section.
Response: Interpretive language was moved to the Discussion section. The Results section now strictly presents findings.

Comment 13: Words such as "prevalence" and "proportion" are used in ways that may be unclear to readers. Use "prevalence" when discussing how widespread a condition is in the population and "proportion" when referring to specific areas of the study sample.
Response: This has been done.

Comment 14: Many p-values are displayed, although it is unclear whether the authors corrected for conducting numerous tests. Explain whether they adjusted for repeated comparisons to prevent false findings.

Response: We did not apply formal corrections for multiple comparisons, as our analyses were based on pre-specified variables informed by existing literature. However, we acknowledge that multiple testing may increase the risk of false-positive findings, and results should be interpreted with caution, especially where p-values are close to the significance threshold. This has been added to the limitation section.

Comment 15: There are various grammatical errors and confusing sentences. Suggestion: Carefully revise the text to improve clarity and professionalism.
Response: The revised manuscript has been extensively reviewed to improve grammar, clarity and professionalism.

Discussion
Comment 1: The discussion part requires further integration and comparison with findings from local and international studies. Currently, the discussion repeats findings without properly contextualizing them within the larger literature on TTIs in Sub-Saharan Africa and related regions.
Please check your prevalence data (particularly the greater syphilis incidence) against national averages or WHO estimates and investigate possible explanations for the discrepancy.
Response: The necessary revisions have been done.

Comment 2: The consequences of high syphilis prevalence are understudied. Are these rates consistent with Ghana's population-level STI trends? Could there be screening concerns (for example, false positives from RDTs).
The discussion of the limitations of rapid diagnostic tests (RDTs) is highlighted but needs to be expanded. You discovered that 7.4% of TTIs were missed by RDTs but later validated by ELISA; this fact requires a more rigorous interpretation and consideration in terms of screening policy.
Response: Thank you for the constructive comments. Your concerns have been duly addressed. We expanded our discussion to reflect that high syphilis seroprevalence may align with broader STI trends in Ghana. We also note that treponemal RDTs, which detect antibodies, cannot distinguish past from active infection, leading to overestimation.

Comment 3: In some cases, the results are merely restated without further analysis. Talking about statistics isn't enough; we also need to look at the reasons behind these trends, particularly about donor types and sociodemographic.

Response: The results obtained from the study have been extensively discussed with the appropriate literature and comparisons.

Conclusion

Comment a: The conclusion includes broad recommendations (e.g., adoption of NAT and policy adjustments to voluntary donation) without taking into consideration feasibility restrictions in low-resource environments.

Response: The characteristics of NAT in terms of unavailability, inaccessibility and high cost make it not feasible currently. Therefore, we recommend it for gradual adoption. These facts have been discussed thoroughly in the revised manuscript.

Comment b: The statement that the findings had "significant implications for hemovigilance" is unclear. Please describe what changes should be made (e.g., prioritizing voluntary donors, discontinuing RDTs, improving donor education, etc.).

Response: We thank the reviewer for highlighting this important point. In the revised Conclusion, we have expanded the implications for haemovigilance to provide specific and actionable recommendations.

Ethical Considerations

Comment: There are a few ethical issues that need to be addressed, including a discrepancy in the ethical approval dates, little information on how donor data was anonymized and protected, and an insufficient explanation for the withdrawal of informed consent.

Response: This study was approved by the Cape Coast Teaching Hospital (CCTH) ethical committee (REF: CCTHERC/EC/2023/174, dated 14th August 2023), amended on 30th January 2025. The study used pre-existing data, and participants were not directly engaged in any phase of the study; therefore, informed consent was not required. Anonymised data were analysed.

All data were handled in compliance with Ghana's patient confidentiality regulations. These have been introduced in the revised manuscript.
There are two approval dates, one was for the main ethical approval and the second one was an amendment to enable us to finalise all the missing or uncaptured data. The two ethical clearance letters were uploaded as supporting files to the journal.

Minor Issues:

Comment 1: The title is clear and informative. Few changes are necessary to comply with PLOS ONE formatting rules, such as uniform formatting of author names, proper use of superscript numbers for affiliations, and limiting the corresponding author to one professional email address.
Response: We have updated the title page to align with PLOS ONE formatting requirements:

Comment 2: Several typographical and grammatical problems can be detected throughout the work; rigorous editing is recommended.
Response: All these errors have been corrected.

Comment 3: Define all abbreviations at the beginning (e.g., NAT, ELISA).
Response: All abbreviations have been defined at first use.

Comment 4: Inconsistencies in statistical reporting (such as p-values) and reference formatting should be addressed.
Response: We reviewed and standardised all statistical reporting to ensure consistency. The reference list formatting has been addressed.

Comment 5: Figures and tables require better formatting, labeling, and the addition of missing information.
Response: All figures and tables have been reformatted for consistency and clarity.

Comment 6: Language usage should be standardized (for example, distinguishing between British and American English).
Response: This has been done.

Comment 7:. The approval timeline looks to be uncertain, anonymization techniques require greater explanation, and the justification for waiving informed consent should be strengthened and related to ethical standards.
Response: This has been done.

Other corrections: Table 3 and the write-up on it have been deleted to ensure conciseness. The previous S1 Table 1 is currently Table 4.

All of the responses and changes have been incorporated into the revised manuscript.

Thank you.
