# Supplementary material for: Prevalence and predictors of transfusion-transmitted infections among blood donor types at a teaching hospital in Ghana: Implications for haemovigilance
Source: PLoS One. 2025 Oct 31;20(10):e0335544. doi: 10.1371/journal.pone.0335544 (PMC12578228; doi:10.1371/journal.pone.0335544)
Supplement: Attachment — Submitted filename: Response to Reviewers.docx [file pone.0335544.s006.docx]

Editor-in-Chief

PLOS ONE

15th August 2025

Dear Editor,

**Re: Revised manuscript submission manuscript number: PONE-D-25-18662R1**

**Title: Prevalence and predictors of transfusion-transmitted infections among blood donor types at a teaching hospital in Ghana: implications for haemovigilance.**

We are sincerely grateful to you and the Reviewers for your time, insightful comments, and constructive feedback, which have greatly improved the quality and rigour of our manuscript. We also thank you for the opportunity to revise and resubmit our work to PLOS ONE.

The manuscript has been carefully revised to address all comments, and our responses are provided point by point. We hope that the revised version meets the requirements for publication in PLOS ONE*,* and appreciate your consideration of our resubmission.

Yours sincerely,

**Dr Alice Charwudzi**
Corresponding author

**Reviewer comments and author responses**

**Journal Requirements:**

**Comment 1:** If the reviewer comments include a recommendation to cite specific previously published works, please review and evaluate these publications to determine whether they are relevant and should be cited. There is no requirement to cite these works unless the editor has indicated otherwise.

**Response:** We did not receive any recommendations from the reviewers or the editor to cite any specific previously published articles.

**Comment 2:** Please review your reference list to ensure that it is complete and correct. If you have cited papers that have been retracted, please include the rationale for doing so in the manuscript text, or remove these references and replace them with relevant current references. Any changes to the reference list should be mentioned in the rebuttal letter that accompanies your revised manuscript. If you need to cite a retracted article, indicate the article’s retracted status in the References list and also include a citation and full reference for the retraction notice.

**Response:** We carefully reviewed the reference list to ensure accuracy and completeness. During revision, some references were reshuffled, citations that were redundant or did not fully support specific statements were removed from those sentences, but retained elsewhere in the manuscript where they remain relevant. All references were verified against PubMed, the respective journal websites, and the Retraction Watch Database (accessed 12 August 2025). None of the cited works had been retracted as of this date.

**REVIEWER #1**

**Comment:** The revised article is deemed satisfactory and acceptable. The modifications implemented have significantly enhanced the overall quality of the study and elevated its scientific rigor. All peer-review concerns have been addressed and revised appropriately. The sole remaining minor issue is the lowercase representation of "n" (denoting number) in Table 4, which should be consistent with the journal's writing guidelines and, if necessary, capitalized.

**Response:** We thank the reviewer for this observation. The “n” has been capitalised in Table 4 per the journal’s style.

**Additional Corrections**

- **Figure 1 Legend:** Revised slightly for clarity. An asterisk was added, and the legend now reads:
  Flowchart of the recruitment and screening process for 10,152 potential blood donors at a teaching hospital in the Central Region of Ghana. ****Donors with incomplete data at initial extraction were excluded without record retention. Of 10,309 captured records, 159 (1.5%) with incomplete fields were removed, leaving 10,152 for analysis.***
- **Table 4 Correction:** On re-checking, we identified a transcription error in the “Prev N (%)” column. The numerators (N) were incorrectly copied, although the percentages and logistic regression outputs (OR, 95% CI, p-values) were based on the correct dataset. The corrected values now align with the denominator (N = 4,451) and percentages. This correction does not affect statistical significance or study conclusions.
- **Proofreading:** We extensively proofread the manuscript, and necessary corrections have been made and tracked.

We believe that the revisions have fully addressed all reviewer and editorial concerns. We thank you again for your consideration.

Thank you.
