# Supplementary material for: Prevalence and predictors of transfusion-transmitted infections among blood donor types at a teaching hospital in Ghana: Implications for haemovigilance
Source: PLoS One. 2025 Oct 31;20(10):e0335544. doi: 10.1371/journal.pone.0335544 (PMC12578228; doi:10.1371/journal.pone.0335544)
Supplement: Attachment — Submitted filename: Response to Reviewers_.docx [file pone.0335544.s007.docx]

Editor-in-Chief

PLoS ONE

15th September 2025

Dear Editor,

**Re: Revised manuscript submission manuscript number: PONE-D-25-18662R2**

**Title: Prevalence and predictors of transfusion-transmitted infections among blood donor types at a teaching hospital in Ghana: implications for haemovigilance.**

We thank the Editor and Reviewers for their constructive feedback, which has helped us strengthen the manuscript. Below, we provide a point-by-point response to each comment, indicating how the manuscript was revised.

We hope that the revised version meets the requirements for publication in PLoS ONE*,* and appreciate your consideration of our resubmission.

Yours sincerely,

**Dr Alice Charwudzi**
Corresponding author

**Reviewer comments and author responses**

**Background**

**Comment 1:** Provide baseline data on Ghana’s TTI prevalence from prior studies (e.g., HBV, HCV, HIV, syphilis rates) to situate your findings in context.

**Response:** We expanded the Introduction **(lines 77-86)** to include prevalence estimates from multiple Ghanaian studies. Studies were selected for methodological robustness and similarity in the methodology in terms of the examined pathogens. Although Hamidu et al. (2023) (<https://doi.org/10.33140/IJHPP.02.02.05>) and Walana et al. (2014) (https://doi.org/10.9734/BMRJ/2014/12160) reported on the prevalence of TTIs in Ghana, they assessed only the viral pathogens, while this present study considered all four major TTIs; thus, they were excluded.

**Comment 2:** Include mention of the window period risk (missed infections due to early-stage infection) as a limitation of RDTs and ELISAs compared to NAT.

**Response:** We revised the Introduction **(lines 97-104)** to highlight that both RDTs and ELISA may miss early infections during the window period, whereas NAT offers superior sensitivity.

**Comment 3:** Add regional context — e.g., "Previous studies in Ghana report TTI prevalence ranging from X–Y%, but reliance on RDTs may underestimate burden."

**Response**: This has been addressed in the Introduction **(lines 77-86 and lines 97-103),** with emphasis on regional prevalence and limitations of RDT-based screening.

**Objective**

**Comment 4:** Refine objective wording for precision: Instead of "assessed the prevalence and predictors of TTIs," specify that you estimated prevalence, identified sociodemographic and donor-type predictors, and compared diagnostic yield of RDTs vs ELISA.
**Response:** The Abstract **(lines 29-31)** and Introduction **(lines 105-109)** now specify that we estimated prevalence, identified sociodemographic and donor-type predictors, and compared the diagnostic yield of RDTs versus ELISA.

**Methods**

**Comment 5:** Clarify the confirmatory testing strategy: Only RDT-non-reactive samples underwent ELISA. Were RDT-reactive samples confirmed with ELISA? If not, prevalence estimates may be inflated due to false positives.

**Response:** We clarified that RDT-reactive samples were not confirmed with ELISA, per national protocol. This is stated in the Method **(lines 172-176),** reiterated in the Discussion **(lines 431-434),** and noted in the Limitations **(lines 530-537)**.

**Comment 6:** Address potential bias between fixed-site and mobile donors: Since the two groups used different screening strategies (RDT+ELISA vs ELISA-only), diagnostic sensitivity may differ, affecting prevalence comparisons.

**Response:** We have now rephrased the portions that acknowledged the differing screening strategies may bias prevalence comparisons (**lines 179-181**), and rephrased the limitation **(lines 533-537).**

**Comment 7:** Specify all covariates included in multivariable regression (e.g., age, sex, education, occupation, donor type, donation frequency) for transparency and reproducibility.

Response: We added the list of covariates (age, gender, donation frequency, donor type and occupation) to the Statistical Analysis section **(lines 217-218).**

**Comment 8:** Define repeat donation criteria clearly (≥2 donations in study period vs. documented donor history).

**Response:** We clarified that repeat donors were defined as individuals with ≥1 prior donation history before the study period (Methods, **lines 136-137**).

**Results/interpretation**

**Comment 9:** Emphasize that donor type was not a significant predictor after adjusting for sociodemographic factors, highlighting confounding effects.

**Response:** This is now emphasised in Results **(lines 344-346)** and Discussion **(lines 466-468).**

**Comment 10:** Discuss RDT false positives: Without confirmatory ELISA for reactive cases, diagnostic accuracy analysis is incomplete.

**Response:** Your concerns were already included in the original manuscript, but we have further done slight rephrasing and introduced additional information in the Methods **(lines 172-176)**, Discussion **(lines 431-434)**, and Limitations **(lines 530-537)**, noting that the lack of an ELISA confirmation for RDT-reactive samples may inflate prevalence estimates.

**Comment 11:** Explore whether fixed-site vs mobile donors differ demographically (age, sex, etc.), since these differences could confound prevalence estimates.

**Response:** We compared the two donor groups and noted key demographic differences in the Results (lines **253-256** and lines **301-304**), and Discussion **(lines 470-472)**. Supporting data are provided in the Supplementary Table (S1B Table).

**Discussion/Conclusion**

**Comment 12:** Add cost and logistical implications of switching from RDTs to ELISA in Ghana, as policymakers will require feasibility considerations.

**Response:** We expanded the discussion (**lines 500-512)** to include cost estimates and logistical considerations for ELISA implementation.

**Comment 13:** Mention NAT (nucleic acid testing) as the gold standard in blood safety. Even if not currently available in Ghana, acknowledging it strengthens the discussion on diagnostic hierarchy.

**Response:** NAT is now acknowledged as the most sensitive method for blood safety in the Introduction **(lines 97-104),** Discussion (**lines 505-512**), Conclusion (**lines 566-567**) and the Abstract’s conclusion **(lines 49-52)**

**Additional corrections**

Some reviewer concerns had already been addressed in the earlier version. To avoid ambiguity, we have rephrased relevant sections to more explicitly align with the feedback. The manuscript has been extensively proofread to remove redundancies and improve clarity. All changes are tracked for transparency.

Thank you.
